# From One Womb to Another: Early Estrogenic Exposures and Later Fibroid Risk

**DOI:** 10.1289/ehp.118-a131a

**Published:** 2010-03

**Authors:** M. Nathaniel Mead

**Affiliations:** **M. Nathaniel Mead**, a science writer living in Durham, NC, has written for **EHP** since 2002

Uterine fibroids (leiomyomata) are the most common pelvic tumors in U.S. women as well as the most common cause for hysterectomy. Both estrogen and progesterone influence fibroid development, whereas early-life hormonal exposures can affect uterine development and a woman’s response to estrogen or progesterone later in life. In a new study, researchers investigate novel hypotheses regarding fibroid pathogenesis in relation to early-life exposures, most of which have not been explored previously **[*EHP*** **118:375–381; D’Aloisio et al.]**.

The authors sought to determine whether *in utero*, early-life, and childhood exposures were linked with self-reported early fibroid diagnosis (by age 35) among non-Hispanic white participants in the NIEHS Sister Study. Participants completed self-administered questionnaires to assess intrauterine and early-life exposures. They also provided information on known and suspected risk factors for breast cancer and other end points including fibroids. The retrospective analysis included nearly 20,000 women who were 35–59 years old when they enrolled in the Sister Study.

The results showed an association between early fibroid diagnosis and having been fed soy formula during infancy, having a mother with pre-pregnancy diabetes, being born at least 1 month early, and low socioeconomic status during childhood. Early fibroid diagnosis also showed associations with prenatal exposure to diethylstilbestrol and with having a mother with gestational diabetes, although these associations were observed only among women who reported probable (versus definite) exposures.

The possibility of long-term health effects of soy formula is of interest because soy contains estrogenic isoflavones, and infants fed only soy formula consume more isoflavones (mostly genistein) per unit body weight than do adults who consume soy foods. The authors report a 25% increase in early fibroid diagnoses for women who had been fed soy formula compared with those who had not. Although the authors postulated the first 2 months of life may include a period more sensitive to isoflavone exposure, they were unable to demonstrate an association with soy formula intake during this time period specifically.

In rodent studies, neonatal treatment with genistein has been associated with later development of uterine cancer, abnormal mammary gland development, differences in hormone receptor levels in mammary glands, altered estrous cycles, reduced fertility, and early reproductive senescence (comparable to menopause in humans). However, there is a lack of human research in this area except for 1 study in which women who had received soy formula as infants reported increased menstrual pain and longer menstrual bleeding (which are symptoms of uterine fibroids).

This also was the first study to evaluate whether *in utero* exposure to maternal diabetes is associated with fibroids. Women whose mothers had diabetes before their pregnancy were twice as likely to report an early fibroid diagnosis as women whose mothers were not diabetic. The authors speculate that *in utero* exposure to maternal diabetes could alter methylation patterns in regions that affect expression of genes relevant to fibroid development.

The main strength of this study is its generation of novel and biologically plausible hypotheses for exploration in future studies. However, because this is the first epidemiologic study to evaluate most of these exposures, replication of findings in other populations is needed.

